# Proteoglycans and neuronal migration in the cerebral cortex during development and disease

**DOI:** 10.3389/fnins.2015.00098

**Published:** 2015-03-23

**Authors:** Nobuaki Maeda

**Affiliations:** Neural Network Project, Department of Brain Development and Neural Regeneration, Tokyo Metropolitan Institute of Medical ScienceSetagaya, Japan

**Keywords:** chondroitin sulfate, extracellular matrix, heparan sulfate, neuronal migration, proteoglycan

## Abstract

Chondroitin sulfate proteoglycans and heparan sulfate proteoglycans are major constituents of the extracellular matrix and the cell surface in the brain. Proteoglycans bind with many proteins including growth factors, chemokines, axon guidance molecules, and cell adhesion molecules through both the glycosaminoglycan and the core protein portions. The functions of proteoglycans are flexibly regulated due to the structural variability of glycosaminoglycans, which are generated by multiple glycosaminoglycan synthesis and modifying enzymes. Neuronal cell surface proteoglycans such as PTPζ, neuroglycan C and syndecan-3 function as direct receptors for heparin-binding growth factors that induce neuronal migration. The lectican family, secreted chondroitin sulfate proteoglycans, forms large aggregates with hyaluronic acid and tenascins, in which many signaling molecules and enzymes including matrix proteases are preserved. In the developing cerebrum, secreted chondroitin sulfate proteoglycans such as neurocan, versican and phosphacan are richly expressed in the areas that are strategically important for neuronal migration such as the striatum, marginal zone, subplate and subventricular zone in the neocortex. These proteoglycans may anchor various attractive and/or repulsive cues, regulating the migration routes of inhibitory neurons. Recent studies demonstrated that the genes encoding proteoglycan core proteins and glycosaminoglycan synthesis and modifying enzymes are associated with various psychiatric and intellectual disorders, which may be related to the defects of neuronal migration.

## Introduction

**Figure 1:**
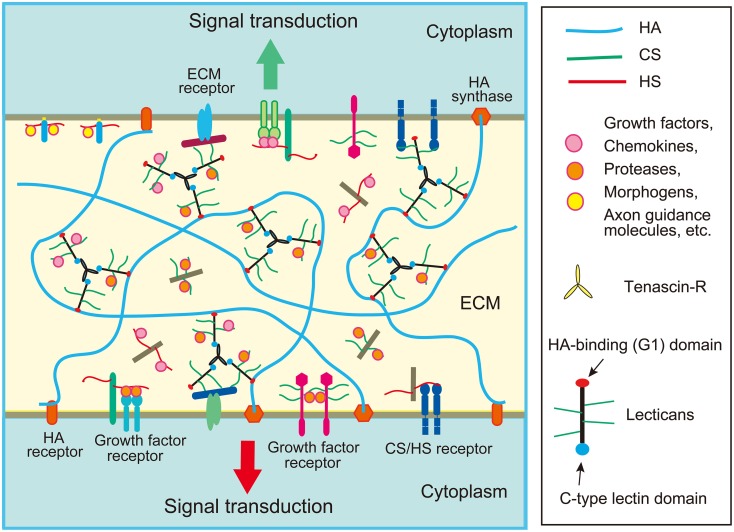
**Schematic structure of extracellular matrix (ECM) in the brain**. The ECM of the brain is mainly composed of chondroitin sulfate (CS) and heparan sulfate (HS) proteoglycans, hyaluronic acid (HA), and glycoproteins such as tenascins. Lectican family CS proteoglycans form large aggregates with HA and tenascins, which store various proteins such as chemokines, growth factors and axon guidance molecules. The ECM proteoglycans may bind with a CS/HS receptor on the cell surface such as RPTPσ. Cell surface proteoglycans may function as receptors or co-receptors for growth factors.

The extracellular matrix (ECM) is a complex network of molecules composed of proteoglycans, hyaluronic acid, fibrous proteins, and various glycoproteins, which fills up the extracellular space within all tissues and organs (Mouw et al., 2014). The ECM also retains water and ions, and constitutes the direct environment surrounding cells, in which multiple types of molecules are cross-linked to each other through protein-protein and protein-carbohydrate interactions, forming the three-dimensional meshworks (Figure 1). The ECM serves not only as a physical scaffold for tissue construction, but also as a dynamic field of signaling that regulates the behavior of cells. In the meshwork of ECM, various signal molecules such as growth factors and chemokines are stored, and the concentration gradients of morphogens such as BMPs and Wnts are also formed. The ECM also serves as an adhesive substrate for the cells, regulating their motility and shape. The structures of ECM are not fixed and static, but are dynamically reorganized by the biosynthesis of its components and their degradation by various proteases and glycanases. Thus, the dynamics of the ECM is quite important in the regulation of cell growth, differentiation, migration, adhesion and tissue morphogenesis.

Unlike other organs, the brain does not normally contain fibrillar collagens except for the basal lamina surrounding the blood vessels and surface of the brain. Instead, major components of brain ECM are proteoglycans. Until recently, many neuroscientists had believed that the brain contains almost no ECM, in spite of the early biochemical and histochemical pioneering work by Margolis et al. (1975, 1976) and Nakanishi (1983), showing the presence of a large amount of glycosaminoglycans and proteoglycans in the developing brain. In the early 1990s, the brain-specific chondroitin sulfate proteoglycans were biochemically characterized (Rauch et al., 1991; Maeda et al., 1992; Oohira et al., 1994), and then their cDNAs were cloned (Rauch et al., 1992; Maeda et al., 1994; Maurel et al., 1994; Watanabe et al., 1995). Then, many investigations revealed the importance of proteoglycans and ECM in the development and disorders of the brain (Franco and Muller, 2011; Maeda et al., 2011; Berretta, 2012; Soleman et al., 2013). However, even now, it seems that the significance of ECM molecules is underestimated in the field of neuroscience, except for reelin. It is desirable that more and more neuroscientists pay attention to the brain ECM molecules.

In this review, I will introduce the structure, binding partners and assembly of proteoglycans and glycosaminoglycans, and discuss their roles in the neuronal migration in the cerebral cortex and their emerging significance in human intellectual disability and psychiatric disorders.

## Assembly of extracellular matrix components

**Figure 2:**
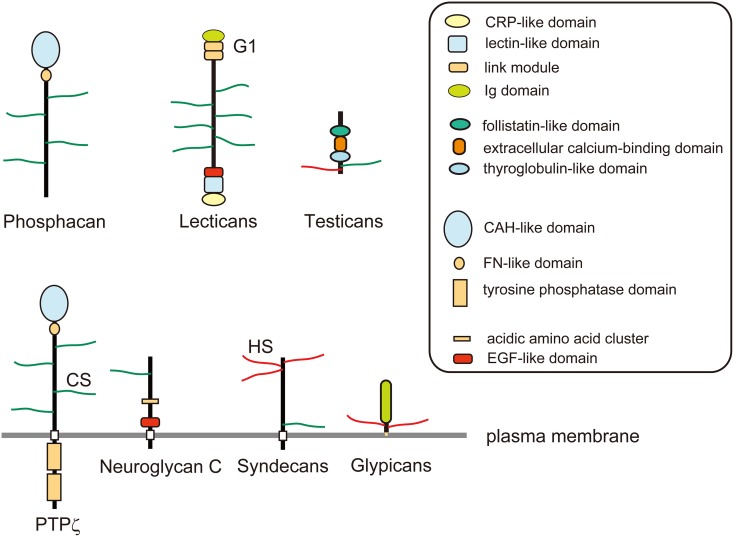
**Schematic structures of proteoglycans**. Phosphacan and lectican family chondroitin sulfate (CS) proteoglycans are major constituents of brain extracellular matrix. PTPζ is a CS proteoglycan-type protein tyrosine phosphatase, which is a splice variant of phosphacan. Neuroglycan C is a transmembrane CS proteoglycan that is classified as Neuregulin-6. Glypicans are a family of glycosyl-phosphatidylinositol (GPI)-anchored heparan sulfate (HS) proteoglycans. Syndecans are a family of transmembrane HS proteoglycans, some members of which may also be modified by CS chains. Testicans are a family of secreted CS/HS proteoglycans.

In the developing cerebral cortex, very high levels of ECM molecules are expressed, the major components of which are chondroitin sulfate proteoglycans. Neurocan, versican, aggrecan, and brevican are lectican family chondroitin sulfate proteoglycans expressed in the brain. Lecticans are secreted proteoglycans that bind with hyaluronic acid through the N-terminal G1 domain that contains an immunoglobulin-like loop and two link modules (Figure 2). These proteoglycans also have a C-type lectin domain at the C-terminal, and chondroitin sulfate chains are covalently attached to the region between the N- and C-terminal domains. The C-type lectin domain of lecticans binds with tenascin family proteins by protein-protein interaction independent of the carbohydrate moiety (Aspberg et al., 1997). Tenascin family proteins are oligomeric glycoproteins with EGF-like repeats and fibronectin-III domains. The brain contains hexameric tenascin-C and trimeric tenascin-R, which promote the assembly of lecticans by protein-protein interaction. On the other hand, hyaluronic acid is a very long polysaccharide consisting of repeating disaccharides of glucuronic acid (GlcA) and *N*-acetylglucosamine (GlcNAc), which are polymerized at the plasma membrane by hyaluronan synthases (HASs). Hyaluronic acids are anchored in the plasma membrane through HASs, or bound to hyaluronan receptors on the cell surface, such as CD44 and RHAMM (Figure 1). The tenascin-lectican complexes bind to the hyaluronic acids through the G1 domains of lecticans, which is stabilized by link proteins (Haplns), forming huge aggregates surrounding cells (Figure 1). It is considered that these huge aggregates serve as a basic framework to construct the ECM in the brain.

Besides secreted chondroitin sulfate proteoglycans, there are also cell surface proteoglycans. PTPζ/RPTPβ and neuroglycan C are major cell surface chondroitin sulfate proteoglycans with a membrane-spanning region. PTPζ is a receptor-type protein tyrosine phosphatase that is synthesized as a chondroitin sulfate proteoglycan (Krueger and Saito, 1992; Maeda et al., 1994). PTPζ has an N-terminal carbonic anhydrase-like domain, a fibronectin-III domain, a membrane-spanning region and two C-terminal tyrosine phosphatase domains. The extracellular domain of this receptor generated by alternative splicing is secreted as a major soluble chondroitin sulfate proteoglycan in the developing brain, phosphacan (Maurel et al., 1994). Phosphacan binds with multiple proteins including pleiotrophin, midkine, tenascins, contactin, and NCAM (Peles et al., 1998). Neuroglycan C is a transmembrane chondroitin sulfate proteoglycan with an EGF module at the juxtamembrane region of the extracellular domain (Watanabe et al., 1995). Chondroitin sulfate-modification of neuroglycan C is developmentally and regionally regulated, and the expression of the non-proteoglycan form increases with development (Aono et al., 2004).

Another major group of proteoglycans in the developing nervous system is heparan sulfate proteoglycans: syndecans and glypicans (Figure 2). The syndecan family is composed of four members, syndecan-1 to -4, each of which has an extracellular domain, a transmembrane region and a conserved short C-terminal cytoplasmic domain (Lambaerts et al., 2009). The N-terminal portion of the extracellular domain of syndecans is modified with heparan sulfate chains. The extracellular domains of syndecan-1 and -4 may be additionally decorated with chondroitin sulfate chains near the transmembrane region (Deepa et al., 2004). It has been considered that most of the extracellular ligand molecules interact with syndecans through binding with heparan sulfate portions. The transmembrane domains of syndecans play important roles in their ligand-induced multimerization and the subsequent signaling (Choi et al., 2005). The intracellular domains of syndecans are divided into two conserved regions (C1 and C2) and a variable region (V), which interact with various kinases and intracellular cytoplasmic components such as src family kinases, CASK, and syntenin (Lambaerts et al., 2009).

Glypicans are glycosyl-phosphatidylinositol (GPI)-anchored heparan sulfate proteoglycans, composed of six family members (glypican-1 to -6) carrying 2–5 heparan sulfate chains (Filmus et al., 2008; Filmus and Capurro, 2014). Glypican-5 was reported also to be modified with chondroitin sulfate in rhabdomyosarcoma cells (Li et al., 2011). The core proteins of glypicans consist of an α-helical domain containing 14 evolutionarily conserved Cys residues, a heparan sulfate-attachment region near the C-terminus, and the C-terminal GPI-anchor attachment signal sequence. Biochemical and genetic studies demonstrated that glypicans bind and regulate Hedgehogs, Wnt, bone morphogenetic proteins and fibroblast growth factors (FGFs) (Filmus and Capurro, 2014). In particular, genetic studies using *Drosophila* demonstrated that glypicans (Dally and Dally-like) play critical roles in the gradient formation of morphogens during wing development (Wu et al., 2010; Raftery and Umulis, 2012). Although it has long been believed that the core proteins of glypicans adopt a globular shape, a recent X-ray crystallographic study indicated that the structure of glypican-1 is actually cylindrical (Svensson et al., 2012).

Testicans are extracellular chondroitin/heparan sulfate proteoglycans, which have been poorly characterized to date. They are composed of three family members (testican-1 to -3), characterized by an N-terminal testican-specific domain, a follistatin-like domain, an extracellular calcium-binding domain, a thyroglobulin-like domain, and a domain with glycosaminoglycan-attachment sites (Schnepp et al., 2005).

## Structure and biosynthesis of chondroitin sulfate and heparan sulfate

**Figure 3:**
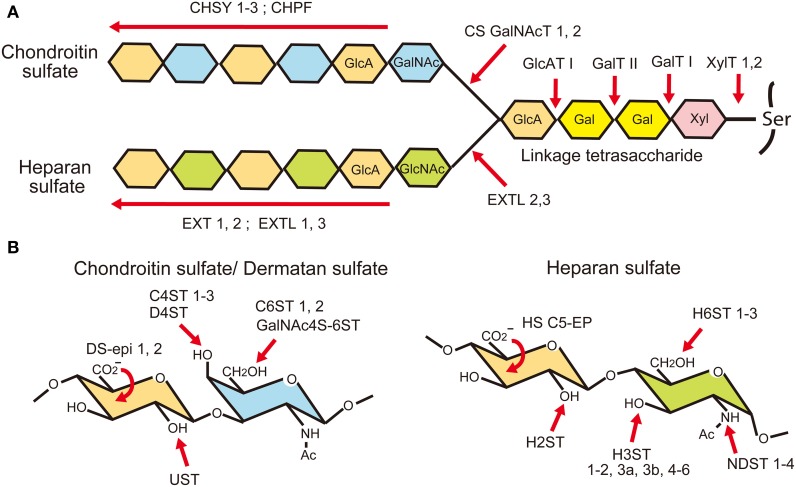
**Biosynthesis of glycosaminoglycans**. **(A)** Chondroitin sulfate (CS) and heparan sulfate (HS) chains are covalently attached to the proteoglycan core proteins through a common linkage tetrasaccharide. It is considered that the biosynthesis of a chondroitin chain starts with the addition of an *N*-acetylgalactosamine (GalNAc) residue to the linkage tetrasaccharide by CS *N*-acetylgalactosaminyltransferases (CSGalNAcT1, 2). After that, glucuronic acid (GlcA) and GalNAc residues are co-polymerized by chondroitin sulfate synthases (CHSY-1, -2, -3) and chondroitin polymerization factor (CHPF). When an *N*-acetylglucosamine (GlcNAc) residue is added to the linkage tetrasaccharide instead of GalNAc by EXTL 2 or 3, a heparan chain is polymerized by EXT family members. **(B)** After polymerization, they are modified by sulfation and epimerization reactions by many glycosaminoglycan modifying enzymes.

Chondroitin sulfate and heparan sulfate are unbranched sulfated polysaccharides covalently attached to the serine residues in proteoglycan core proteins via common linkage tetrasaccharides, GlcAβ1-3galactose(Gal)β1-3Galβ1-4xylose(Xyl)β1-O-Ser (Figure 3). Chondroitin sulfate is composed of repeating disaccharide units of *N*-acetylgalactosamine (GalNAc) and GlcA, (GlcAβ1-3GalNAcβ1-4)n, whereas heparan sulfate is composed of repeating disaccharide units of GlcNAc and GlcA, (GlcAβ1-4GlcNAcα1-4)n. Biosynthesis of these polysaccharides is initiated by the addition of Xyl residues to the specific serine residues in core proteins by xylosyl transferases (XYLT1, 2) (Nadanaka and Kitagawa, 2008; Mikami and Kitagawa, 2013; Mizumoto et al., 2013a). This is followed by the addition of two Gal residues and a GlcA residue by galactosyltransferase-I (B4GALT7), galactosyltransferase-II (B3GALT6) and glucuronyltransferase-I (B3GAT3), respectively. After that, repeating disaccharide units of chondroitin or heparan sulfate are polymerized in the Golgi apparatus. Chondroitin sulfates are polymerized by chondroitin sulfate synthases (CHSY-1 to -3), chondroitin polymerization factor (CHPF) and chondroitin sulfate *N-acetylgalactosaminyltransferases* (CSGalNAcT-1, -2), whereas heparan sulfates are polymerized by EXT family members (EXT1, EXT2, EXTL1, EXTL2, EXTL3). Since these glycosaminoglycans use a common linkage tetrasaccharide, the chain initiation step determines whether a chondroitin or a heparan chain elongates. If the first GalNAc residue is added to the linkage tetrasaccharide possibly by CSGalNAcT-1 or -2, a chondroitin chain is elongated by the CHSY/CHPF complex. If a GlcNAc residue is alternatively added to the linkage tetrasaccharide possibly by EXTL 2 or 3, a heparan chain is polymerized by the EXT1/EXT2 complex. Although the genes encoding these biosynthetic enzymes have been identified, almost nothing is known about the mechanism for the selection of glycosaminoglycan types at the chain initiation step.

After the polymerization of repeating disaccharides, they are heavily modified by the C5 epimerization of GlcA residues and sulfation reactions (Nadanaka and Kitagawa, 2008; Mikami and Kitagawa, 2013; Mizumoto et al., 2013a) (Figure 3). The majority of the GalNAc residues in chondroitin sulfate are 4-*O-sulfated* by chondroitin 4-*O*-sulfotransferases (C4ST-1 to -3) or 6-*O*-sulfated by 6-*O*-sulfotransferases (C6ST-1 and -2). Although many of the disaccharide units in chondroitin sulfate are mono-sulfated A units [GlcAβ1-3GalNAc(4-SO_4_)] or C units [GlcAβ1-3GalNAc(6-SO_4_)], they may be further sulfated by GalNAc 4-sulfate 6-*O*-sulfotransferase (GalNAc 4S-6ST) or chondroitin uronyl 2-*O*-sulfotransferase (UST), generating di-sulfated disaccharides, E units [GlcAβ1-3GalNAc(4, 6-bis-SO_4_)] or D units [GlcA(2-SO_4_)β1-3GalNAc(6-SO_4_)], respectively. Furthermore, some of the GlcA residues are C5-epimerized to iduronic acid (IdoA) by dermatan sulfate epimerases 1 and 2 (encoded by *DSE* and *DSEL*, respectively). The resulting IdoAα1-3GalNAc units are sulfated by dermatan 4-*O*-sulfotransferase (D4ST), generating an iA unit [IdoAα1-3GalNAc(4-SO_4_)], which may be further sulfated by UST, generating an iB unit [IdoA(2-SO_4_)α1-3GalNAc(4-SO_4_)]. Chondroitin sulfate with a high content of IdoA is often called dermatan sulfate.

The modification of heparan sulfate begins with the *N-sulfation* reaction by *N*-deacetylase/*N*-sulfotransferase (NDST-1 to -4), which removes acetyl groups from some of the GlcNAc residues in the heparan sulfate chain and replaces them with sulfate groups (Figure 3). Then, some of the GlcA residues are C5 epimerized to IdoA by heparan sulfate glucuronyl C5-epimerase (HS C5-EP), followed by *O*-sulfation reactions. The *O*-sulfation includes 2-*O*-sulfation of GlcA/IdoA residues by heparan sulfate 2-*O*-sulfotransferase (H2ST), 3-*O*-sulfation of glucosamine (GlcN) units by heparan sulfate 3-*O*-sulfotransferases (H3ST-1, -2, -3A, -3B, -4, -5, and -6), and 6-*O*-sulfation of GlcN units by heparan sulfate 6-*O*-sulfotransferases (H6ST-1 to -3). Because *N*-sulfation of GlcN units by NDSTs generates substrates for the subsequent modification enzymes, highly *N*-sulfated regions in heparan sulfate are also highly modified by C5-EP and various *O*-sulfotransferases. Thus, heparan sulfate chains display domain structures: highly modified NS-domains, poorly modified NA-domains characterized by stretches of *N*-acetylated disaccharide units, and the interspacing NA/NS-domains composed of both *N*-acetylated and *N*-sulfated disaccharide units.

## Binding partners of glycosaminoglycans

Recent studies using microarrays and surface plasmon resonance revealed that chondroitin sulfate and heparan sulfate chains bind with many proteins that play important roles in brain development, especially neuronal migration (Deepa et al., 2002; Kawashima et al., 2002; Maeda et al., 2006; Shipp and Hsieh-Wilson, 2007; Conrad et al., 2010; Rogers et al., 2011; Mizumoto et al., 2013b). Both heparan sulfate and chondroitin sulfate chains bind with various axon guidance molecules in a sulfation pattern-dependent manner (Shipp and Hsieh-Wilson, 2007). While Slit2 shows a preference for heparan sulfate sequences that contain 6-*O*-sulfation and *N*-sulfation, netrin 1 requires sulfation at the 2-*O*-, 6-*O*-, and *N*-positions. Semaphorin5B (Sema5B), ephrinA1 and ephrinA5 prefer 2-O- and *N*-sulfation. On the other hand, all of these axon guidance molecules bind strongly with E unit-rich highly sulfated chondroitin sulfate E from squid cartilage (CS-E) (Shipp and Hsieh-Wilson, 2007). Sema5B also binds moderately with D unit-rich shark cartilage chondroitin sulfate D (CS-D), and weakly with A unit-rich whale cartilage chondroitin sulfate A (CS-A), pig skin dermatan sulfate (CS-B) and C unit-rich shark cartilage chondroitin sulfate C (CS-C). EphrinA1 binds moderately with CS-C, and weakly with CS-B and CS-D. Slit2, netrin1 and ephrinA5 bind only weakly with CS-A, -C, -D, and -B. In addition, it has been reported that Sema3A binds strongly with CS-E (Dick et al., 2013). EphrinA3 also binds with heparan sulfate and chondroitin sulfate, although the structural requirement is unknown (Irie et al., 2008; Conrad et al., 2010).

Neurotrophin family growth factors [nerve growth factor (NGF), brain-derived neurotrophic factor (BDNF), neurotrophin-3 (NT-3), and neurotrophin-4 (NT-4)] bind strongly with CS-E (Rogers et al., 2011). BDNF, NT-3, and NT-4, but not NGF bind moderately with CS-A, whereas CS-C shows almost no binding to these proteins. PC12 cells express E unit-rich chondroitin sulfate, and its removal upon chondroitinase ABC treatment significantly attenuated TrkA activation by NGF or NT-4, suggesting that endogenous chondroitin sulfate plays important roles in neurotrophin signaling (Rogers et al., 2011). Glial cell line-derived neurotrophic factor (GDNF) associates with heparan sulfate in a 2-*O*-sulfation-dependent manner, promoting the binding of this protein to its receptor component GFRα1 (Rickard et al., 2003).

Chemokines are a family of small proteins that induce chemotaxis of various cells including cortical interneurons (Marin, 2013). Glycosaminoglycans interact with chemokines such as CCL2 (MCP-1), CCL5 (RANTES), and CXCL12 (SDF-1) in a chain length- and sulfation pattern-dependent manner (Kuschert et al., 1999; Hirose et al., 2001). Among the chemokines, CXCL12 plays important roles in the tangential migration of cortical interneurons (see below). CXCL12 binds strongly with CS-E and the highly sulfated S domains in heparan sulfate, and also interacts weakly with CS-A, -B, -C, and -D (Murphy et al., 2004; Mizumoto et al., 2013b). In addition, it was revealed that versican interacts with CXCL12 through chondroitin sulfate chains (Hirose et al., 2001). It has been considered that these interactions in the ECM and cell surface contribute to the formation of immobilized or haptotactic gradients of chemokines (Kuschert et al., 1999).

Pleiotrophin and midkine are a family of multifunctional heparin-binding growth factors that bind with both heparan sulfate and chondroitin sulfate (Perez-Pinera et al., 2007; Muramatsu, 2014). These growth factors bind strongly with highly sulfated heparan sulfate and CS-E, moderately to CS-B and CS-D, and very weakly to CS-A (Maeda et al., 2003; Zou et al., 2003; Mizumoto et al., 2013b).

The structures of glycosaminoglycans are determined at least partly by the combinatorial expression of their modifying enzymes. During development of the brain, the sulfotransferases involved in the chondroitin/heparan sulfate synthesis show dynamic spatiotemporal expression patterns (Yabe et al., 2005; Mitsunaga et al., 2006; Ishii and Maeda, 2008b). This suggests that the expression of specific functional domains in these glycosaminoglycan chains is strictly regulated in the developing brain. Conway et al. (2011) showed that the expressions of *HS2ST* and *HS6ST-1* are distinctively regulated at the optic chiasm, and the mutant mice lacking these genes exhibit different axon guidance defects. Interestingly, *HS2ST*^−/−^ and *HS6ST-1*^−/−^ phenotypes closely match those of *Slit1*^−/−^ and *Slit2*^−/−^, respectively, suggesting that slit family proteins are regulated by specific sulfation of heparan sulfate.

## Ligand binding to proteoglycans

As described above, glycosaminoglycans bind with various protein ligands in a structure-dependent manner. However, the ligand binding to proteoglycans is extremely complex because many proteoglycans carry multiple glycosaminoglycan chains that may function cooperatively. Furthermore, cooperation between the core protein and attached glycosaminoglycan chains may also occur. Accordingly, proteoglycans usually exhibit much higher affinity and/or avidity for the ligand proteins than free glycosaminoglycans (Herndon et al., 1999). Thus, degradation of proteoglycan core proteins by extracellular proteases may terminate such cooperativity and release the ligand molecules, leading to the activation or inactivation of the signaling in a context-dependent manner. Cooperation is observed not only between glycosaminoglycans of the same type but also between heparan sulfate and chondroitin sulfate chains. Syndecan-1 and syndecan-4 carry both heparan sulfate and chondroitin sulfate chains, which cooperatively regulate the binding dynamics of pleiotrophin, midkine and FGF-2 to these proteoglycans (Deepa et al., 2004).

PTPζ/phosphacan binds to pleiotrophin and midkine, in which both chondroitin sulfate and core protein portions contribute to the interaction (Maeda et al., 1996, 1999, 2003). While intact phosphacan preparation shows low (*Kd* = 3 nM) and high affinity binding (*Kd* = 0.25 nM) for pleiotrophin, this proteoglycan exhibits only single very low affinity binding after chondroitinase ABC-treatment (*Kd* = 13 nM) (Maeda et al., 1996). This suggests that the binding affinity of phosphacan for pleiotrophin is regulated by the structural variation of chondroitin sulfate. In fact, the structure of chondroitin sulfate on phosphacan changes during rat brain development, and a slight increase in the content of oversulfated D unit drastically strengthens the binding of this proteoglycan to pleiotrophin (Maeda et al., 2003).

Another prominent example of the cooperation between the core protein and glycosaminoglycan chain in the signaling has been reported by the group of Filmus (Li et al., 2011; Filmus and Capurro, 2014). They revealed that glypican-3 and glypican-5 oppositely regulate the Hedgehog (Hh) signaling in rhabdomyosarcoma cell proliferation. Glypican-3 binds to Hh through its core protein, reducing the amount of Hh available to its receptor Patched 1 (Ptc1), with the consequent decrease in signaling. On the other hand, glypican-5 interacts with both Hh and Ptc1 through heparan sulfate and chondroitin sulfate chains, facilitating Hh-Ptc1 binding with the consequent increased signaling. The heparan sulfate chains of glypican-5 show a higher degree of sulfation than those of glypican-3, which may explain why the glycosaminoglycan chains of glypican-5 but not those of glypican-3 interact with Hh/Ptc1.

Sema5A is an axon guidance molecule that can exert both inhibitory and permissive effects on growing axons. Kantor et al. (2004) revealed that Sema5A interacts with the glycosaminoglycan portion of both chondroitin sulfate and heparan sulfate proteoglycans. The axonal heparan sulfate proteoglycans are required for the Sema5A-mediated attraction of growing axons of the fasciculus retroflexus. On the other hand, the extracellular chondroitin sulfate proteoglycans precisely localize Sema5A in a specific area, where Sema5A acts as a repulsive guidance cue for these growing axons. Thus, the bifunctional roles of Sema5A are regulated by chondroitin sulfate and heparan sulfate proteoglycans, demonstrating the cooperation among these two types of proteoglycans during the process of axon pathfinding.

Recently, Coles et al. (2011) reported that receptor protein tyrosine phosphatase σ (RPTPσ) is a receptor for both chondroitin sulfate and heparan sulfate proteoglycans. Heparan sulfate proteoglycans induce oligomerization of RPTPσ on the growth cone, leading to inactivation of the tyrosine phosphatase activity and growth promotion. On the other hand, extracellular chondroitin sulfate proteoglycans inhibit oligomerization of this receptor with consequent suppression of axon growth. It is considered that multiple RPTPσ molecules bind to the islands of high/intermediate sulfation on heparan sulfate chains (NS to NA/NS domains), which stabilize the receptor oligomers. Conversely, the receptor binding sites are considered to be sparsely distributed on chondroitin sulfate chains, and therefore receptor oligomerization cannot occur. If so, the signaling of RPTPσ should be highly dependent on the glycosaminoglycan structures. Proteoglycans bearing low sulfated heparan sulfate chains may inhibit receptor oligomerization, and conversely proteoglycans bearing highly sulfated chondroitin sulfate may induce receptor oligomerization. It is also reported that another receptor tyrosine phosphatase, leukocyte common antigen-related phosphatase (LAR) is a functional receptor for chondroitin sulfate proteoglycan (Fisher et al., 2011).

Although the structure of glycosaminoglycans is basically determined by the biosynthetic processes, the sulfation pattern of heparan sulfate may be modified extracellularly by the endosulfatases, Sulf1 and Sulf2 (Nagamine et al., 2012). These sulfatases catalyze the desulfation of the 6-*O*-sulfate group from GlcN residues in the trisulfated disaccharides in heparan sulfate. Thus, highly sulfated NS domains in heparan sulfate are preferentially desulfated by these enzymes, leading to the change in the binding affinity of various ligands to heparan sulfate proteoglycans. This results in the activation or suppression of specific signaling molecules such as Wnt, GDNF, and FGF during various developmental processes (Ai et al., 2003, 2007; Wang et al., 2004). Thus, functions of proteoglycans are intricately regulated at multiple levels.

## Roles of proteoglycans in neuronal migration

**Figure 4:**
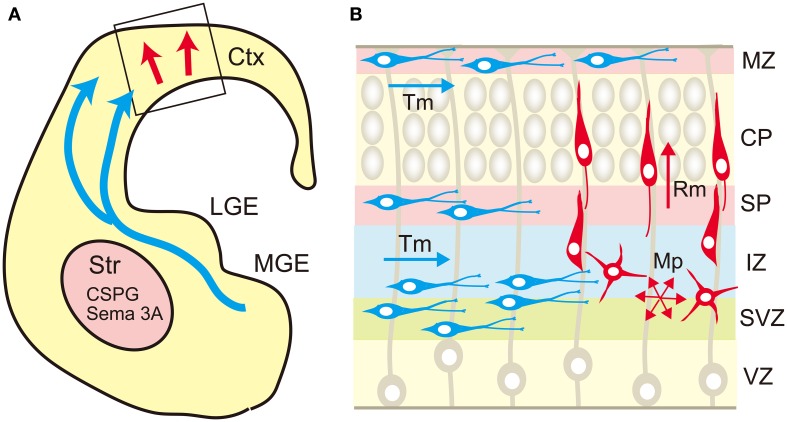
**Migration routes of excitatory and inhibitory neurons in the cerebrum**. **(A)** The excitatory neurons are generated in the ventricular zone of the neocortex (Ctx) and migrate radially toward the brain surface (red arrow). The cortical inhibitory neurons are generated mainly in the medial ganglionic eminence (MGE), and migrate tangentially toward the neocortex (blue arrows). The migrating interneurons avoid the striatum (Str) that expresses chondroitin sulfate proteoglycans (CSPG) and semaphorin 3A (Sema 3A). **(B)** In the neocortex, the excitatory neurons (red cells) born in the ventricular zone (VZ) show multipolar morphology and migrate in random directions (Mp) in the subventricular (SVZ) and intermediate (IZ) zones. When the multipolar neurons reach the subplate (SP), they transform into a bipolar shape and migrate radially (Rm) in the cortical plate (CP) toward the marginal zone (MZ). On the other hand, the tangential migration (Tm) of interneurons (blue cells) occurs in a layer-specific manner, in which interneurons prefer MZ, SP, lower IZ, and SVZ.

**Figure 5:**
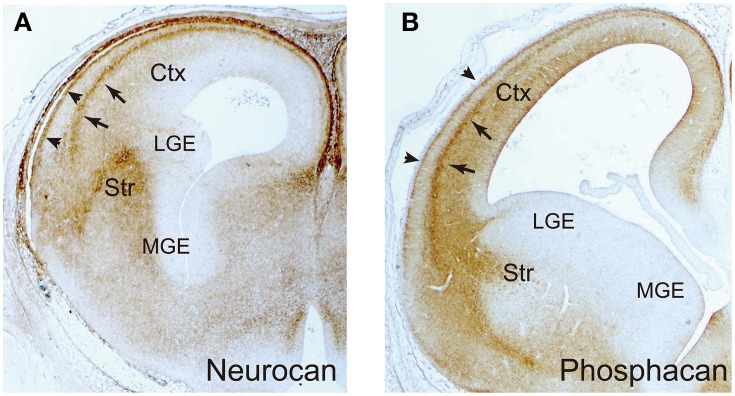
**Immunohistochemical localization of neurocan and phosphacan**. The frontal sections of embryonic day 16 rat brains were immunohistochemically stained with anti-neurocan **(A)** and anti-phosphacan **(B)** monoclonal antibodies. They are selectively expressed in the marginal zone (arrowheads) and subplate (arrows) in the neocortex, and the striatum (Str). The medial (MGE) and lateral (LGE) ganglionic eminences are negative.

In the developing neocortex, postmitotic pyramidal neurons generated in the ventricular zone show a multipolar shape, and migrate in random directions in the subventricular and intermediate zones (Figure 4). After that, they transform into the bipolar shape and attach to the radial glial fibers, upon which they rapidly migrate toward the marginal zone. This multipolar-to-bipolar transition occurs when the neurons reach the subplate, suggesting that this layer contains critical factor(s) regulating neuronal behavior (Ohtaka-Maruyama et al., 2013). The radial migration of pyramidal neurons stops at the interface between the cortical plate and the marginal zone, forming the “inside-out” arrangement of neurons. On the other hand, inhibitory neurons tangentially migrate in the neocortex through the marginal zone, subplate, and lower intermediate/subventricular zones. These migration patterns of both excitatory and inhibitory neurons suggest that specific cortical layers play critical roles in the regulation of neuronal migration. Nakanishi (1983) demonstrated that glycosaminoglycans stained by colloidal iron distributed principally in the marginal zone and subplate in the developing mouse cerebral cortex. In the cortices of reeler mutants, where radial migration of pyramidal neurons is severely disturbed, most glycosaminoglycans are localized in the outer layer of the cortex. From such an expression pattern, it was suggested that glycosaminoglycans are involved in the neuronal migration and/or laminar pattern formation of the neocortex. Then, the brain-specific chondroitin sulfate proteoglycans were identified, and it was revealed that neurocan and phosphacan are richly expressed in the marginal zone and subplate (Oohira et al., 1994; Maeda et al., 1995; Meyer-Puttlitz et al., 1996) (Figure 5). Versican was also localized in these layers (Popp et al., 2003), raising the possibility that chondroitin sulfate proteoglycans regulate neuronal migration in the cortex.

As described above, PTPζ/phosphacan binds to pleiotrophin with high affinity (Maeda et al., 1996, 1999). Pleiotrophin induces oligomerization of PTPζ, which leads to the inactivation of its tyrosine phosphatase activity, initiating downstream signaling (Meng et al., 2000; Fukada et al., 2006). Pleiotrophin is deposited along radial glial fibers, and PTPζ is expressed on the migrating pyramidal neurons, raising the possibility that pleiotrophin on radial glial fibers regulates the radial migration of excitatory neurons (Maeda and Noda, 1998). In fact, *in vitro* cell migration assay demonstrated that pleiotrophin-PTPζ signaling induces migration of cortical neurons (Maeda and Noda, 1998). Subsequently, this signaling system has been demonstrated to promote the migration of various types of normal and tumor cell in a chondroitin sulfate-dependent manner (Polykratis et al., 2005; Feng et al., 2010; Koutsioumpa et al., 2013). Phosphacan and free chondroitin sulfate suppress the pleiotrophin-induced neuronal migration by competitive inhibition of the binding between pleiotrophin and cell surface PTPζ (Maeda and Noda, 1998; Maeda et al., 1999). Soluble chondroitin sulfate proteoglycans such as phosphacan, neurocan and versican expressed in the subplate and marginal zone may regulate the migratory behavior of neurons by inhibiting pleiotrophin- PTPζ signaling.

Recently, it was found that neuroglycan-C is involved in the radial migration of pyramidal neurons from a study of the plant homeodomain (PHD) finger 6 (PHF6) gene (Zhang et al., 2013). *PHF6* is an X-linked gene encoding the protein that has four nuclear localization signals and two PHD-type zinc finger domains, which functions as a transcription repressor. Mutations of PHF6 cause Börjeson-Forssman-Lehmann syndrome, characterized by intellectual disability associated with seizures, short stature, hypogonadism, hypometabolism, marked gynecomastia, truncal obesity, tapered fingers, narrow palpebral fissure, and large ears (Liu et al., 2014). Using *in utero* electroporation, Zhang et al. (2013) demonstrated that knockdown of PHF6 severely impaired the radial migration of cortical neurons. They also identified neuroglycan-C as a downstream target of PHF6. Knockdown of neuroglycan-C phenocopied the neuronal migration phenotype of PHF6 knockdown, suggesting that PHF6 controls the expression level of neuroglycan-C in the cortical neurons, thus regulating the radial neuronal migration. Neuroglycan C binds with pleiotrophin and midkine, in which its chondroitin sulfate portion increases the affinity of the core protein for these growth factors (Ichihara-Tanaka et al., 2006; Nakanishi et al., 2010). There is a possibility that pleiotrophin/midkine-neuroglycan C signaling is involved in the radial migration in the cerebral cortex.

Pleiotrophin also binds with syndecan-3 in a heparan sulfate-dependent manner (Raulo et al., 1994; Kinnunen et al., 1996). Like PTPζ, syndecan-3 is required for pleiotrophin-induced neuronal migration, suggesting that PTPζ and syndecan-3 are redundant pleiotrophin receptors on cortical neurons (Hienola et al., 2006). Syndecan-3 knockout mice showed delayed radial neuronal migration in the cortex, and this delay was partially caught up at ~10 days after birth (Hienola et al., 2006). The binding of pleiotrophin to syndecan-3 triggers the phosphorylation of src, which then activates cortactin and modulates the assembly of the actin cytoskeleton. Syndecan-3 knockout mice also show migration defects of interneurons (Bespalov et al., 2011). At embryonic day 15, calbindin-positive precursors of interneurons were accumulated in the ganglionic eminence of syndecan-3 knockout mice, and the density of GABA-immunoreactive cells was lower in the dorsomedial cortex of adult knockout mice than in that of control mice. It has been suggested that GDNF binds to the heparan sulfate portion of syndecan-3 on interneurons, promoting their migration. On the other hand, syndecan-1 is expressed by the neural progenitor cells in the cerebral cortex (Wang et al., 2012). Knockdown of syndecan-1 using *in utero* electroporation resulted in the reduction of neural progenitor cells and the promotion of neuronal differentiation in the cortex (Wang et al., 2012). In these cortices, there were fewer cells in the ventricular/subventricular zone, and more neurons moved into the intermediate zone and cortical plate compared with the cortices electroporated with control plasmid. These findings suggest that syndecan-1 and syndecan-3 are differentially expressed and play distinct roles in the developing cortex.

## Roles of glycosaminoglycans in neuronal polarization

During development of the mouse cerebrum, the disaccharide composition of chondroitin sulfate changes dynamically (Ishii and Maeda, 2008a). At embryonic day 16 (E16), the major components are A and C units, and then the content of A unit increases and that of C unit decreases until maturation. A small but significant amount of E unit is detected at E16 to 18, and the content decreases thereafter. D unit is a minor component, but is constantly detected during embryonic and postnatal development. Since D and E units contribute significantly to the binding of various ligand molecules as described above, we investigated the roles of these oversulfated structures in neuronal migration (Ishii and Maeda, 2008a). Using *in utero* electroporation, we introduced shRNA constructs for GalNAc 4S-6ST and UST into neural progenitor cells in the ventricular zone of the E14 cortex. At E18, the embryos were dissected out, and the migration of the cortical neurons was examined. Knockdown of both sulfotransferases severely disrupted the radial migration of cortical neurons. The neurons knocked down for these enzymes were accumulated in the subventricular zone and in the intermediate zone, and showed multipolar morphology. This suggested that oversulfated chondroitin sulfate is required for the multipolar-to-bipolar transition of pyramidal neurons.

Neuronal polarization of dissociated hippocampal pyramidal cells is a well-established *in vitro* model of multipolar-to-bipolar transition of newborn neurons (Dotti et al., 1988). Dissociated hippocampal pyramidal neurons extend several morphologically indistinguishable minor processes several hours after plating. Then, one of these minor processes extends rapidly and becomes an axon, and the other processes differentiate into dendrites. In contrast, the hippocampal neurons cultured in the presence of chondroitinase ABC extended multiple axon-like processes that were highly unstable and repeatedly extended and retracted (Nishimura et al., 2010). The morphology and behavior of the chondroitinase ABC-treated neurons were similar to those of multipolar neurons in the developing cortex. Furthermore, knockdown of GalNAc 4S-6ST and UST also disturbed the neuronal polarization of cultured hippocampal neurons, suggesting the importance of oversulfated chondroitin sulfate in this process. In the cultured hippocampal neurons, the oversulfated chondroitin sulfate was accumulated in the focal contacts in the cell bodies and axons. Chondroitinase ABC-treatment suppressed the tyrosine phosphorylation of FAK at the focal contacts, suggesting that the proteoglycans bearing oversulfated chondroitin sulfate strengthen the adhesion of axons and cell bodies to the substrate, leading to the stabilization of neuronal morphology. In contrast to the chondroitinase ABC-treatment, the axons extended steadily and showed almost no retraction when hippocampal neurons were treated with heparitinases that specifically degrade heparan sulfate. This suggests that heparan sulfate proteoglycans destabilize the neuronal morphology, inducing retraction of axons. In fact, it has been reported that heparan sulfate proteoglycans on growth cones are essential for the repulsive activities of Slit2 and ephrin-A3 (Hu, 2001; Irie et al., 2008). Thus, chondroitin sulfate and heparan sulfate proteoglycans expressed on hippocampal neurons play opposing roles during neuronal polarization.

## Roles of glycosaminoglycans in tangential neuronal migration

Cortical interneurons are born in the medial ganglionic eminence (MGE), caudal ganglionic eminence and preoptic area in the ventral telencephalon, and migrate tangentially toward the cortex (Evsyukova et al., 2013; Marin, 2013) (Figure 4). The newborn interneurons exiting the ganglionic eminence avoid entering the striatum, and migrate into the neocortex through the marginal zone, subplate, or lower intermediate/subventricular zones, suggesting that complex interplay of repulsive and attractive cues regulates the migration route of these neurons. Neuregulin-1, NT-4, and GDNF were shown to be chemoattractive factors for cortical interneurons, whereas Slit 1, Sema 3A, ephrin a3, and ephrin a5 act as chemorepulsive factors (Zhu et al., 1999; Polleux et al., 2002; Flames et al., 2004; Rudolph et al., 2010; Bespalov et al., 2011; Marin, 2013; Steinecke et al., 2014). As described above, biochemical studies revealed that these factors bind with chondroitin and/or heparan sulfate in a sulfation pattern-dependent manner. Thus, there is a possibility that chondroitin/heparan sulfate proteoglycans regulate the spatial distribution and/or activity of these factors. In fact, a recent report demonstrated that chondroitin sulfate plays an important role in the tangential migration of interneurons (Zimmer et al., 2010). Chondroitin sulfate proteoglycans are highly expressed in the striatal mantle zone, which is avoided by tangentially migrating interneurons (Figure 5). *In vitro* Boyden chamber cell migration and stripe assays demonstrated that chondroitin sulfate proteoglycans exert repulsive effects on cortical interneurons. These repulsive effects were suppressed by chondroitinase ABC-treatment, suggesting that chondroitin sulfate directly acts as a repellent for these neurons. Furthermore, in the chondroitinase ABC-treated brain slices, cortical interneurons actively invaded the striatum, although they avoided this region in the control slices. Sema3A is retained in the striatum by binding to the chondroitin sulfate chains, and repels migrating interneurons that express Sema3A receptor, neuropilin 1. Thus, it was shown that chondroitin sulfate proteoglycans exert not only direct, but also indirect repulsive effects on interneurons by anchoring repulsive factors in the striatum (Figure 4).

After the interneurons enter the neocortex, they avoid the cortical plate, where chondroitin sulfate proteoglycans are poorly expressed (Figure 4). Instead, they migrate through the chondroitin sulfate proteoglycan-rich marginal zone and subplate. They also prefer the subventricular zone and the lower intermediate zone, where the content of chondroitin sulfate proteoglycans is relatively low. Thus, it seems that chondroitin sulfate proteoglycans do not act as a repellent for interneurons in the neocortex. Tangential migration of interneurons in the neocortex is induced by the chemokine CXCL12 (SDF1), which is concentrated in the marginal zone, subplate, and lower intermediate/subventricular zones (Li et al., 2008; Lopez-Bendito et al., 2008). It has been reported that CXCL12 binds with both chondroitin sulfate and heparan sulfate (Mbemba et al., 2000). The marginal zone and subplate are highly enriched with phosphacan, neurocan and versican (Oohira et al., 1994; Maeda et al., 1995; Meyer-Puttlitz et al., 1996) (Figure 5), and thus CXCL12 may be anchored to the chondroitin sulfate chains of these proteoglycans. On the other hand, syndecan-1 is highly expressed in the ventricular/subventricular zone and in the lower intermediate zone (Wang et al., 2012), and may concentrate CXCL12 in these layers through heparan and/or chondroitin sulfate moieties. Thus, it seems that proteoglycans can be either attractive or repulsive substrates depending on the proteins bound to their glycosaminoglycan chains.

As described above, syndecan-3 functions as a GDNF receptor expressed on migrating interneuron (Bespalov et al., 2011). In this case, only the matrix-bound form of GDNF acts as a ligand of syndecan-3, and the soluble form is not active. It may be that GDNF bound to the chondroitin/heparan sulfate proteoglycans in the extracellular matrix activates the syndecan-3 signaling in the interneurons.

## Proteoglycans, psychiatric disorders, and intellectual disabilities

Since proteoglycans are major components of the connective tissue, mutations in the proteoglycan-related genes cause various skeletal and connective tissue disorders (Huegel et al., 2013; Mizumoto et al., 2013a). Recently, the involvement of these genes in intellectual and psychiatric disorders has also begun to be revealed (Tables 1, 2). A hypofunctional mutation of *XYLT1* encoding xylosyltransferase 1 causes an autosomal recessive short stature syndrome associated with intellectual disability (Schreml et al., 2014). Mutations of *B3GALT6* encoding galactosyltransferase II cause a pleiotropic Ehlers-Danlos-syndrome-like connective tissue disorder, which is also associated with intellectual disability (Malfait et al., 2013). As described above, these two enzymes are involved in the biosynthesis of the linkage tetrasaccharides that are used commonly for the chain initiation of chondroitin and heparan sulfates, implying that these glycosaminoglycans are essential for the development of higher intellectual function of the brain. In fact, an earlier study suggested that *EXT1*, encoding a heparan sulfate co-polymerase, is associated with autism (Li et al., 2002). Loss-of-function mutations in *CHSY1* encoding chondroitin synthase 1 cause Temtamy preaxial brachydactyly syndrome, which is characterized by delayed motor and mental development as well as bilateral, symmetric preaxial brachydactyly and hyperphalangism of digits, facial dysmorphism, and dental anomalies (Li et al., 2010). Furthermore, it was found that missense mutations of *NDST1* cause intellectual disability, muscular hypotonia, and epilepsy, suggesting that normal modification of heparan sulfate is essential for the development of functional neuronal circuits (Reuter et al., 2014).

**Table 1:** **Human disorders caused by mutations of proteoglycan-related genes**.

**Genes (coded proteins)**	**Clinical features**	**References**
*XylT1* (Xylosyltransferase 1)	Autosomal recessive short stature syndrome; distinct facial features, alteration of fat distribution, intellectual disability	Schreml et al., 2014
*B3GALT6* (Galactosyltransferase II)	Pleiotropic Ehlers-Danlos-syndrome-like connective tissue disorder; skin fragility, delayed wound healing, joint hyperlaxity, and contractures, muscle hypotonia, spondyloepimetaphyseal dysplasia, intellectual disability	Malfait et al., 2013.
*CHSY1* (Chondroitin synthase 1)	Temtamy preaxial brachydactyly syndrome; bilateral preaxial brachydactyly and hyperphalangism of digits, facial dysmorphism, dental anomalies, sensorineural hearing loss, intellectual disability	Li et al., 2010.
*NDST1* (NDST1)	Intellectual disability, muscular hypotonia, epilepsy, postnatal growth deficiency	Reuter et al., 2014.
*SPOCK1* (Testican-1)	Intellectual disability, partial agenesis of corpus callosum, prenatal-onset microcephaly, artrial septal defects	Dhamija et al., 2014.
*GPC3* (Glypican 3)	Simpson-Golabi-Behmel syndrome type I; pre/postnatal overgrowth, distinctive craniofacial features, macrocephaly, organomegaly. Intellectual disability and epilepsy in some cases	Tenorio et al., 2014.

**Table 2:** **Proteoglycan-related genes proposed to be associated with mental disorders**.

**Genes (coded proteins)**	**Mental disorders**	**References**
*DSEL* (DS epimerase 2)	Bipolar disorder, depressive disorder	Goossens et al., 2003; Shi et al., 2011
*UST* (UST)	Job-related exhaustion	Sulkava et al., 2013
*NDST3* (NDST3)	Schizophrenia, bipolar disorder	Lencz et al., 2013
*EXT1* (EXT1)	Autism	Li et al., 2002
*NCAN* (Neurocan)	Schizophrenia, bipolar disorder	Muhleisen et al., 2012; Schultz et al., 2014
*PTPRZ1* (Phosphacan/PTPζ)	Schizophrenia	Buxbaum et al., 2008; Takahashi et al., 2011
*CSPG5* (Neuroglycan C)	Schizophrenia	So et al., 2010

In addition to glycosaminoglycan biosynthetic enzymes, genes encoding proteoglycan core proteins are also associated with intellectual disability. Missense mutation in *SPOCK1* encoding testican-1 causes intellectual disability with dyspraxia, dysarthria, partial agenesis of corpus callosum, and prenatal-onset microcephaly (Dhamija et al., 2014). Simpson-Golabi-Behmel syndrome is an overgrowth/multiple congenital anomalies syndrome caused by mutations in *glypican 3*. This disease shows high clinical variability, and in some cases, intellectual disability is present (Tenorio et al., 2014). It will be important to examine whether these intellectual disabilities are caused by abnormal neuronal migration in the cortex.

It has recently been revealed that early developmental defects of neural network formation including abnormal neuronal migration and myelination can cause various psychiatric diseases such as schizophrenia (Stolp et al., 2012). Muhleisen et al. (2012) identified variation in the neurocan gene (rs1064395) as a common risk factor for bipolar disorder and schizophrenia. In schizophrenia patients, *neurocan* risk status was found to be associated with higher folding in the right lateral occipital cortex and left dorsolateral prefrontal cortex (Schultz et al., 2014). Neurocan may play important roles in the neuronal migration and/or formation of axonal fibers in the cerebral cortex, and the deficits in these processes may influence the folding of the occipital and prefrontal lobes, leading to an increased risk of schizophrenia.

It is well known that several members of the Neuregulin/ErbB signaling system are susceptibility genes of schizophrenia, bipolar disorders and depression (Mei and Nave, 2014). Neuregulins constitute a family of EGF-like signaling molecules that stimulate ErbB receptor family tyrosine kinases, the signaling of which regulates neuronal migration, myelination, neurotransmission and synaptic plasticity (Mei and Nave, 2014). Recent studies suggested that proteoglycans regulate Neuregulin/ErbB signaling, and thus are related to psychiatric disorders. Buxbaum et al. (2008) reported that *PTPRZ1*, which encodes both PTPζ and phosphacan, is associated with schizophrenia in a Caucasian population, although no association was found in the Japanese population (Ito et al., 2008). They demonstrated that PTPζ binds with ErbB4 through the scaffolding protein, MAGI, and inhibits the Neuregulin-1/ErbB4 signaling. Furthermore, Takahashi et al. (2011) found that the expression of PTPζ is increased in the brains of schizophrenia patients, and also demonstrated that transgenic mice overexpressing PTPζ showed reduced Neuregulin-1 signaling, and abnormal glutamatergic, GABAergic and dopaminergic activity as well as delayed oligodendrocyte development. In particular, it is remarkable that the number of parvalbumin-positive interneurons is decreased in the cortex of this transgenic mouse. Flames et al. (2004) demonstrated that loss of Neuregulin-1/ErbB4 signaling causes an alteration in the tangential migration of cortical interneurons and reduction in the number of GABAergic interneurons in the postnatal cortex. Therefore, PTPζ/phosphacan may negatively regulate Neuregulin-1/ErbB4 signaling, and inhibit the tangential migration of cortical interneurons.

*Neuroglycan C* was identified as a potential susceptibility gene for schizophrenia in a Southern Chinese population (So et al., 2010). As described above, neuroglycan C is involved in the radial neuronal migration in the neocortex (Zhang et al., 2013), and thus defects in this process may be involved in the etiology of schizophrenia. In addition, neuroglycan C has an EGF-like domain, acts as a direct ligand for ErbB3, and thus is classified as Neuregulin-6 (Kinugasa et al., 2004). It has been reported that ErbB3 is associated with schizophrenia in a Caucasian population (Li et al., 2009), and thus there is a possibility that neuroglycan C-ErbB3 signaling is involved in the pathophysiology of schizophrenia. ErbB3 plays important roles in oligodendrocyte differentiation and myelination (Mei and Nave, 2014), and neuroglycan C may regulate these processes. In this context, it is interesting to note that midkine-neuroglycan C signaling promotes process elongation of the oligodendrocyte precursor-like cell line, CG-4 (Ichihara-Tanaka et al., 2006).

In addition to proteoglycan core proteins, glycosaminoglycan-modifying enzymes have also been associated with psychiatric disorders. A genome-wide association study revealed that *NDST3* is associated with schizophrenia and bipolar disorder, suggesting that the sulfation pattern of heparan sulfate plays an important role in the pathophysiology of these disorders (Lencz et al., 2013). Neuregulin-1 binds with heparan sulfate in a sulfation pattern-dependent manner, in which the *N*-sulfate group is the most important (Pankonin et al., 2005). Thus, the *N*-sulfated region in heparan sulfate may be important for normal Neuregulin-1/ErbB4 signaling.

The genomic region containing *DSEL* encoding dermatan sulfate epimerase 2 has been found to be associated with bipolar disorder (Goossens et al., 2003) and depressive disorder (Shi et al., 2011). In addition, it was reported that *UST* is associated with job-related exhaustion and response to antidepressant (Uher et al., 2010; Sulkava et al., 2013). Thus, chondroitin sulfate proteoglycans bearing oversulfated dermatan/chondroitin sulfate may play important roles in the etiology of mood disorders.

## Perspective

As described above, brain proteoglycans regulate the migration of both excitatory and inhibitory neurons by binding with various proteins. Besides neuronal migration, proteoglycans play important roles in the proliferation and differentiation of neural progenitor cells, axon pathfinding, myelination, axon regeneration, and maturation and plasticity of synapses (Maeda et al., 2011; Soleman et al., 2013; Silver and Silver, 2014; Theocharidis et al., 2014), the defects of which may be related to the pathogenesis of various brain disorders. More mechanistic studies are necessary to elucidate the relationship between proteoglycans and these diseases. In this context, it should be noted that many extracellular matrix proteins that interact with proteoglycans are overlooked in the field of developmental neuroscience. These include extracellular matrix proteases and their inhibitors such as MMP, ADAMTS, ADAM, and Timp family members. Proteoglycans turn over very rapidly in the developing brain, and their degradation would lead to drastic change in the distribution and activity of growth factors, chemokines, axon-guidance molecules, and so on. Thus, it is likely that degradation of proteoglycans would profoundly influence the behavior of neurons. Future study is necessary to shed light on this issue.

Finally, I would like to emphasize that the functions of proteoglycans are regulated in a context-dependent manner. It is often said that chondroitin sulfate proteoglycans are repulsive molecules. However, this over-simplified view has been challenged by the finding that chondroitin sulfate proteoglycans such as PTPζ and neuroglycan C promote the radial migration of cortical neurons. Furthermore, chondroitin sulfate proteoglycans may function as either a repulsive or an attractive substrate depending on the factors attached to the chondroitin sulfate chains. In addition, it should be noted that even though the proteoglycan core protein is the same, the structures of the attached glycosaminoglycan chains may be highly variable leading to the diversification of proteoglycan functions. I expect that careful experimental design and interpretation of the results would uncover the important functions of brain proteoglycans.

### Conflict of interest statement

The authors declare that the research was conducted in the absence of any commercial or financial relationships that could be construed as a potential conflict of interest.

## Abbreviations

BDNF: brain-derived neurotrophic factor
C4-ST: chondroitin 4-*O*-sulfotransferase
C6-ST: chondroitin 6-*O*-sulfotransferase
CHPF: chondroitin polymerization factor
CHSY: chondroitin synthase
CSGalNAcT: chondroitin sulfate *N*-acetylgalactosaminyltransferase
E: embryonic day
Gal: galactose
GalNAc: *N*-acetylgalactosamine
GalNAc 4S-6ST: *N*-acetylgalactosamine 4-sulfate 6-*O*-sulfotransferase
GlcA: glucuronic acid
GDNF: glial cell line-derived neurotrophic factor
GlcN: glucosamine
GlcNAc: *N*-acetylglucosamine: GPI, glycosylphosphatidylinositol
ECM: extracellular matrix
HAS: hyaluronan synthase
Hh: hedgehog
HS: heparan sulfate
H2ST: heparan sulfate 2-*O*-sulfotransferase
H3ST: heparan sulfate 3-*O*-sulfotransferase
H6ST: heparan sulfate 6-*O*-sulfotransferase
HS C5-EP: heparan sulfate C5-epimerase
IdoA: iduronic acid
LAR: leukocyte common antigen-related phosphatase
NDST: *N*-deacetylase/*N*-sulfotransferase
NGF: nerve growth factor
NT: neurotrophin
ptc: patched
PHF: plant homeodomain finger
PHD: plant homeodomain
Sema: semaphorin
UST: uronyl 2-*O*-sulfotransferase
Xyl: xylose.
